# Highly Selective Ratiometric Sensors for Pb^2+^ Based on Luminescent Zn(II)-Coordination Polymers with Thiophenedicarboxylate. Crystal Structures and Spectroscopic Studies

**DOI:** 10.1007/s10895-024-03754-1

**Published:** 2024-05-28

**Authors:** Georgina M. Otero-Fuentes, Victor Sánchez-Mendieta, Alejandro Sánchez-Ruiz, Raúl A. Morales-Luckie, Diego Martínez-Otero, Jonathan Jaramillo-García, Juan Pablo León-Gómez, Alejandro Dorazco-González

**Affiliations:** 1 Facultad de Química, Universidad Autónoma del Estado de México, Paseo Colón y Paseo Tollocan, Toluca, Estado de México 50120 México; 2 Centro Conjunto de Investigación en Química Sustentable UAEM-UNAM, Carretera Toluca‑Atlacomulco Km. 14.5, San Cayetano, Toluca, Estado de México 50200 México; 3 Institute of Chemistry, National Autonomous University of Mexico, Circuito Exterior, Ciudad Universitaria, Ciudad de México, 04510 México; 4 Tecnológico Nacional de México, Campus Zitácuaro (ITZ), Av. Tecnológico 186, Colonia Manzanillos, 61534 H. Zitácuaro, Michoacán México

**Keywords:** Zn(II) coordination polymers, 2,5-thiophenedicarboxylate, Luminescent sensors, Pb^2+^ selective sensing

## Abstract

The development of luminescent coordination polymers for the selective sensing of Pb^2+^ in water constitutes an active area of research that impacts analytical, environmental, and inorganic chemistry. Herein, two novel water-stable 2D Zn-coordination polymers {[Zn_2_(H_2_O)_2_(tdc)_2_(bpy)]·(H_2_O)}_n_ **1** and [Zn(tdc)(tmb)]_n_ **2** (tdc = thiophenedicarboxylate; bpy = 4,4’-bipyridine and tmb = 4,4'-trimethylenebipyridine) were synthesized, structurally determined by single crystal X-ray diffraction, and studied in-depth as luminescent sensors for a series of cations (Ca^2+^, Mg^2+^, Mn^2+^, Fe^2+^, Co^2+^, Ni^2+^, Cu^2+^, Zn^2+^ Cd^2+^, Hg^2+^ and Pb^2+^) in 20% aqueous ethanol. These Zn-polymers possess photostability in 20% aqueous ethanol with a strong emission at 410 upon excitation at 330 nm and quantum yields of around Φ = 0.09. Under these conditions, Pb^+2^ can be efficiently sensed with polymer** 2** through a fluorescent ratiometric response with selectivity over common interfering metal ions such as Cu^2+^, Cd^2+^ and Hg^2+^ in the micromolar concentration range (detection limit = 1.78 ± 10 μM). Such selectivity/affinity of Pb^2+^ over Hg^2+^ for luminescent chemosensors is still rare. On the basis of spectroscopic tools (^1^H NMR, far ATR-IR, PXRD), the X-ray crystal structure of **2**, and Scanning Electron Microscopy with Energy-Dispersive X-ray Spectroscopic analysis, the ratiometric fluorescent response is proposed via an efficient metal-ion exchange driven through interactions between thiophenedicarboxylate rings and Pb^2+^ ions. The use of flexible luminescent Zn-coordination polymers as sensors for selective and direct detection of Pb^2+^ in aqueous media has been unexplored until now.

**Graphical Abstract:**

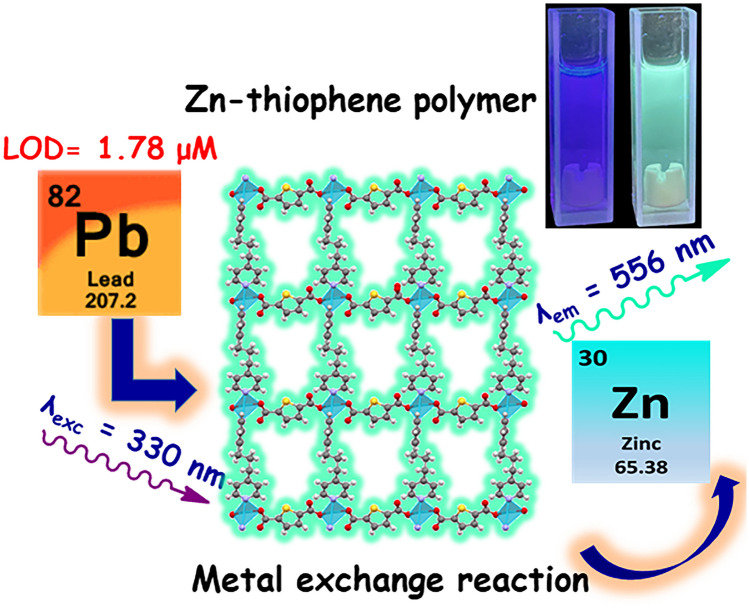

**Supplementary Information:**

The online version contains supplementary material available at 10.1007/s10895-024-03754-1.

## Introduction

Luminescence coordination polymers (LCPs) continue demonstrating their structural versatility and usefulness as efficient sensing materials towards numerous different chemical substances in diverse environments [1–3]. The main advantages of using LCPs as probes are their relatively easy synthesis, their chemical structure versatility, and their sensitivity and selectivity for diverse chemicals such as anions, cations, biochemical substances, toxic pollutants, among others [4–8]. It seems that the main downfall in the use of these hybrid materials as luminescent sensors continue being their lack of stability in aqueous media [7]. Nonetheless, the study of novel LCPs is mainly driven by the almost endless variety of chemical structures, the growing interest of developing efficient, rapid, and as low-cost as possible analytical tools for the detection of chemical substances in many research and technological areas, and the inherent necessity of acquiring further knowledge to surmount those breakdowns in this field. Particularly, Zn(II)-LCPs have considerably been studied as sensors of various chemical compounds ranging from heavy metal ions and organic molecules [9] to drugs [10]. Among the Zn(II)-LCPs reported, there are various assembled with ligands that include a sulfur moiety in their chemical structure: 2,5-tiophenedicarboxylate [11], 4,4’-dipyridylsulfide [12], mercaptonicotinate [13] and thiosemicarbazone of glyoxalic acid [14] are some of the main reported examples of these type of ligands. The use of 2,5-tiophenedicarboxylate (tdc) as a bridging ligand of Zn(II)-CPs has been reported in several cases; however, most of the studies deal with the structural characterization, solid-state luminescence properties, catalytic properties or gas sorption experiments [15–19]. Solid-state photoluminescence studies of a series of Zn(II) 3-D CPs having tdc and bis(imidazole) derivative ligands, emission maxima of the polymers were obtained in a range from 442 to 460 nm, upon excitation at 380 nm; these emissions were attributed to ligand-to-metal charge transfer (LMCT), in addition to the rigidity and asymmetry of the tdc ligand [20]. Furthermore, luminescent sensing studies have also been reported for some Zn(II)-LCPs bearing the tdc bridging ligand; the 2D MOF {[Zn(tdc)(3-abit)]·H_2_O}_n_, (3-abit = 4-amino-3,5-bis(imidazol-1-ylmethyl)-1,2,4-triazole) exhibited good selective sensing properties for CrO_4_^2−^; Cr_2_O_7_^2−^ in water [21]; also, the CP {[Zn(L)(tdc)⋅2H_2_O]_2_}_n_ (L = 1,3-bis(benzimidazol-1-yl)-2-propanol) showed highly selective and sensitivity for luminescence sensing of acetone and Cr_2_O_7_^−2^ anions in aqueous solution [11].

Detection of Pb^2+^ in several media is relevant due to the human health implications, and damages to the environment, that this heavy-metal ion can produce [22]. Some LCPs have been employed in studies of Pb^2+^ sensing, bearing metal ions such as Co(II) [23], Cd(II) [24], Ag(I) [25], Y(III) [26], Tb(III) [27], among other lanthanides [28]. Moreover, although certain Zn(II) LCPs have been reported for the sensing of Pb^2+^ [29–32], these Zn-complexes are not particularly selective and common interfering cations such as Hg^2+^ and Cd^2+^ can be a problem.

Actually, in a recent review about Zn(II)/Cd(II) LCPs as sensors of metal ions, there are only two Cd(II) LCPs described as efficient and highly-selective Pb^2+^ ions detectors, but none based on Zn(II) [7].

Herein, the Zn(II)-CPs: {[Zn_2_(H_2_O)_2_ (tdc)_2_(bpy)]·(H_2_O)}_n_ **1** and [Zn(tdc)(tmb)]_n_ **2** were synthesized by facile self-assembly reactions, structurally characterized, and their luminescent sensing properties towards metal ions were studied in aqueous ethanolic dispersions.

## Experimental

### General Conditions

All the materials were of reagent grade purchased from commercial sources and used without further purification. De-ionized water was used for synthetic procedures. Elemental analyses for C, H and N were carried out by standard methods using a Vario Micro-Cube analyzer. IR spectra of crystalline polymers were determined with a Bruker Tensor 27 spectrophotometer, with Platinum ATR, in the range 4000 to 400 cm^−1^. Other ATR-IR spectra were recorded on a FT-IR NICOLET IS-50 spectrophotometer. ^1^H NMR spectra were performed on a Bruker Advance 300 MHz spectrometer. The chemical shifts (δ) are given in ppm relative to TMS as internal standard.

### Synthetic Procedures

Zn-polymer** 1**. Sodium 2,5-thiophenedicarboxylate was initially prepared by adding an aqueous solution (5 ml) of NaOH (0.004 g; 0.1 mmol) to a methanol solution (5 ml) of 2,5-thiophenedicarboxylic acid (0.0086 g; 0.05 mmol). Then, 4,4’-bipyridine (0.0078 g; 0.05 mmol), in 5 mL of MeOH, was incorporated to the previous solution, and the resulted mixture was added to an aqueous solution (5 ml) of ZnCl_2_ (0.0136 g; 0.1 mmol). The translucent solution obtained was allowed to evaporate slowly; after four days, off-white crystals were attained, which were filtered, washed with methanol and water, and air-dried. Elemental analysis (%), C_22_H_22_N_2_O_13_S_2_Zn_2_, cal.: 36.83 C, 3.09 H, 3.90 N; found: 36.79 C, 3.06 H, 3.93 N. IR (ATR, cm^−1^) (Fig. S1): 3094(m, br); 1607(m); 1524(s); 1411(m), 1352(s); 1222(m); 1118(w); 1065(w); 1020(w); 846(w); 773(s); 723(m); 630(s); 462(s).

Zn-polymer** 2**. The same conditions of the synthesis of **1** were used, except that a solution of 4,4'-trimethylenebipyridine (0.0198 g; 0.05 mmol) in MeOH (10 ml) was added to the sodium 2,5-thiophenedicarboxylate solution while stirring. After three days, off-white crystals were obtained. Elemental analysis (%), C_19_H_16_N_2_O_4_SZn, cal.: 52.60 C, 3.72 H, 6.46 N; found: 52.43 C, 3.76 H, 6.25 N. IR (ATR, cm^−1^) (Fig. S2): 3077(vw); 2944(vw); 2862(vw); 1632(m); 1619(m); 1594(m); 1528(m); 1431(m), 1374(s); 1325(m); 1301(s); 1229(m); 1107(w); 1068(w); 1029(m); 840(w); 813(m); 797(m); 767(s); 684(m); 615(m); 576(m); 524(s); 464(m).

#### Crystallographic Analysis

Single-crystal X-ray diffraction studies were performed on a Bruker Smart Apex Duo diffractometer equipped with an Apex II CCD detector at 100 K using Mo–Ka radiation (k = 0.71073 Å) from an Incoatec ImuS source and a Helios optic monochromator. Suitable crystals were coated with hydrocarbon oil, picked up with a nylon loop, and mounted in the cold nitrogen stream of the diffractometer. Frames were collected using ω scans and integrated with SAINT [33]. Multiscan absorption correction (SADABS) was applied [33]. The structures were solved by direct methods and refined using full-matrix least-squares on F2 with SHELXL-2018 [34] using the SHELXLE GUI [35]. Weighted R factors, Rw, and all goodness-of-fit indicators, are based on F2. All non-hydrogen atoms were refined anisotropically. The hydrogen atoms of the C − H bonds were placed in idealized positions; whereas in **1**, the hydrogen atoms from the OH moieties in the water molecules were localized from the difference electron density map and their position was refined with Uiso tied to the parent atom with distance restraints. Also, the zinc atom in **1** exhibits disorder that was modeled in two positions with different compositions, the first position was modeled as zinc atom bonded to two molecules of water in the axial positions of a trigonal bipyramid geometry, while in the second position, only one molecule is bonded to zinc atom collocated at the apex of a tetrahedral geometry in the zinc atom, the ratio of the occupancy was fixed in 0.5 for each part in the disorder. The disordered groups were refined without restrictions of geometry for found the correct positions of the atoms and using Uij restraints (SIMU, RIGU, EADP) implemented in SHELXL-2018 [34]. In **2**, the carbon and one oxygen atoms of one carboxylate moiety of the tdc ligand exhibit disorder that was modeled in two positions with ratio 57/43. The disordered moiety was refined using geometry (SADI, SAME, FLAT) and Uij restraints (SIMU, RIGU) implemented in SHELXL-2018 [34].

Crystallographic data for **1** and **2** have been deposited at the Cambridge Crystallographic data Center (CCDC) with the deposition numbers 2163979 and 2163980, respectively. The relevant details of the crystals, data collection and structural refinement can be found in Table S1. The selected bond distances, bond angles and hydrogen bonds are specified in Tables S2 and S3.

#### Luminescence Measurements

Photoluminescence spectra for solid-state samples and aqueous ethanolic dispersions of **1** and **2** were recorded on an Agilent Cary Eclipse fluorescence spectrophotometer, which features a Xenon lamp and is equipped with a holder for crystals and a cell thermostat holder with a quartz cuvette. Crystalline samples of **1** and **2** were used for luminescence experiments upon excitation at 330 nm. Luminescent titration experiments were carried out by adding aliquots of aqueous concentrated stock solutions of the salts M(NO_3_)_2_ (50 mM) or Pb(NO_3_)_2_ (5 mM) to aqueous dispersions (EtOH-H_2_O, 8/2, v/ v) of **1** or **2** (10 µM). After each addition, the dispersion was equilibrated for 2.0 min before recording the luminescence spectrum (λ_ex_ = 330 nm) using a quartz cuvette. Stock ethanolic dispersions of polymers **1** and **2** were prepared by stirring 1.0 mg of the corresponding polymer in 2.0 mL of ethanol for 10 min at 25 °C to give a final concentration of 0.70 mM and 1.15 mM for **1** and **2**, respectively. Luminescence quantum yields (Φ) were determined using an aqueous solution of quinine sulphate in H_2_SO_4_ (0.5 M) as a standard (Φ = 0.546; λ_ex_ = 360 nm). For the determination of the quantum yield, the excitation wavelength was chosen so that A < 0.05 [10].

## Results and Discussion

### Structural Description

Polymer **1** crystallizes in the triclinic space group P-1. The asymmetric unit includes one Zn(II), one tdc ligand, one bpy ligand, one aqua ligand and one lattice water molecule.

**Fig. 1:**
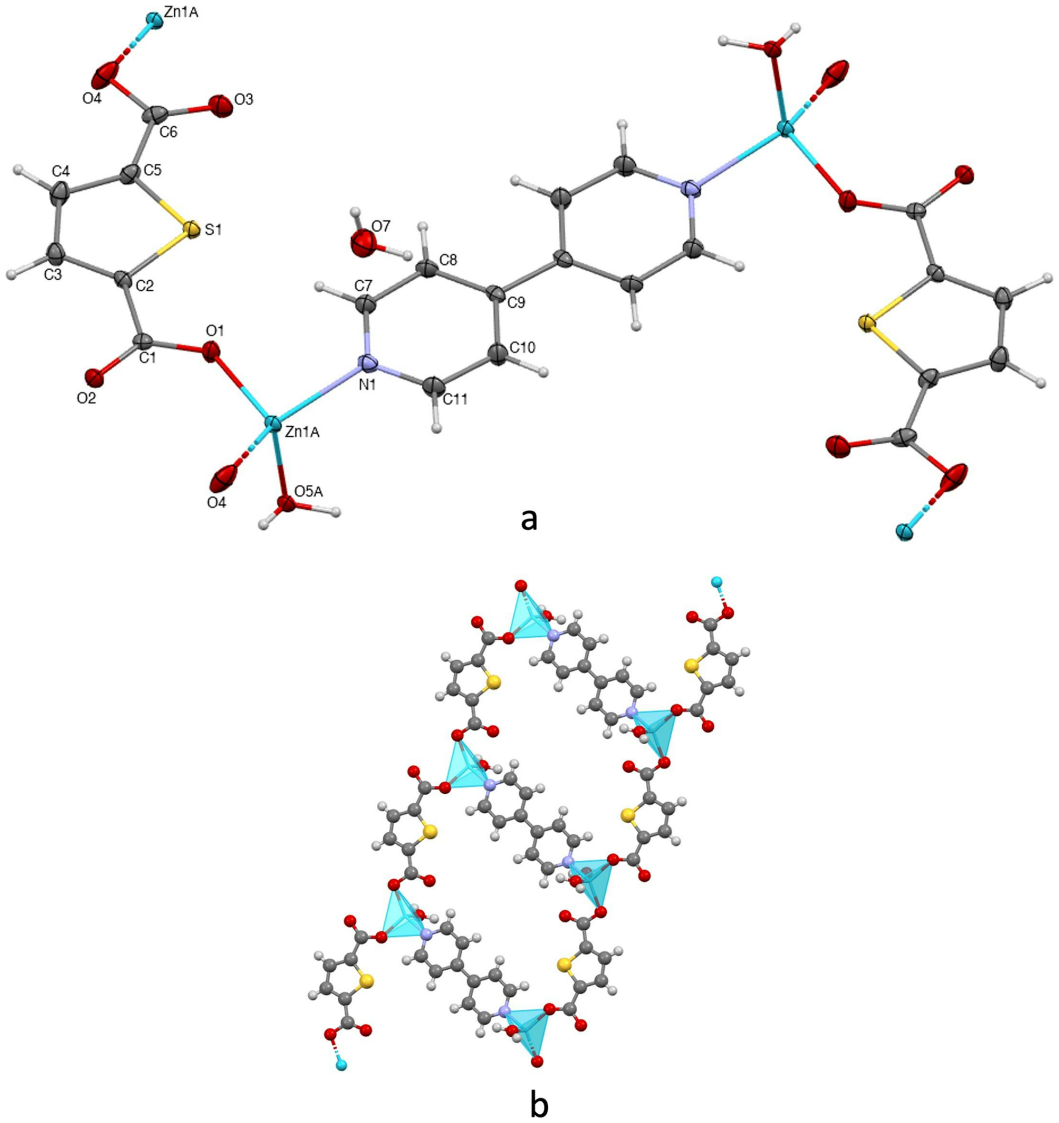
Molecular structure of **1** (ellipsoids shown at 60% probability) (**a**). 1D array of **1**; view down the *c* axis (**b**)

The four-coordinated Zn(II) is surrounded by two oxygens from two tdc ligands, one oxygen from the aqua ligand and one nitrogen from the bpy, exhibiting a distorted tetrahedral coordination geometry. In the molecular structure of **1**, there are two Zn(II) ions bridged by a bpy ligand, and two tdc ligands are attached to those metal centers (Fig. 1a). The Zn–O and Zn-N distances and, O–Zn–O and O-Zn-N bond angles ranging from 1.9385(16) Å to 2.0794(17) Å and from 95.11(7)° to 134.95(7)°, respectively, are comparable to those found in related reported four-coordinated Zn(II) complexes [16, 18, 36]. In **1**, the tdc ligand appears in a bridging monodentate coordination mode, while the bpy ligand participates with its typical *trans* bridging monodentate coordination mode. Thus, the combination of those coordination modes around the Zn(II) centers yield a double-ion 2D with a stepladder structure (Fig. 1b). These chains are further connected throughout hydrogen bonds involving the non-bonded carboxylate oxygens of tdc, the aqua ligand and the lattice water molecule (Table S2), generating thus a 3D supramolecular structure (Fig. S3).

**Fig. 2:**
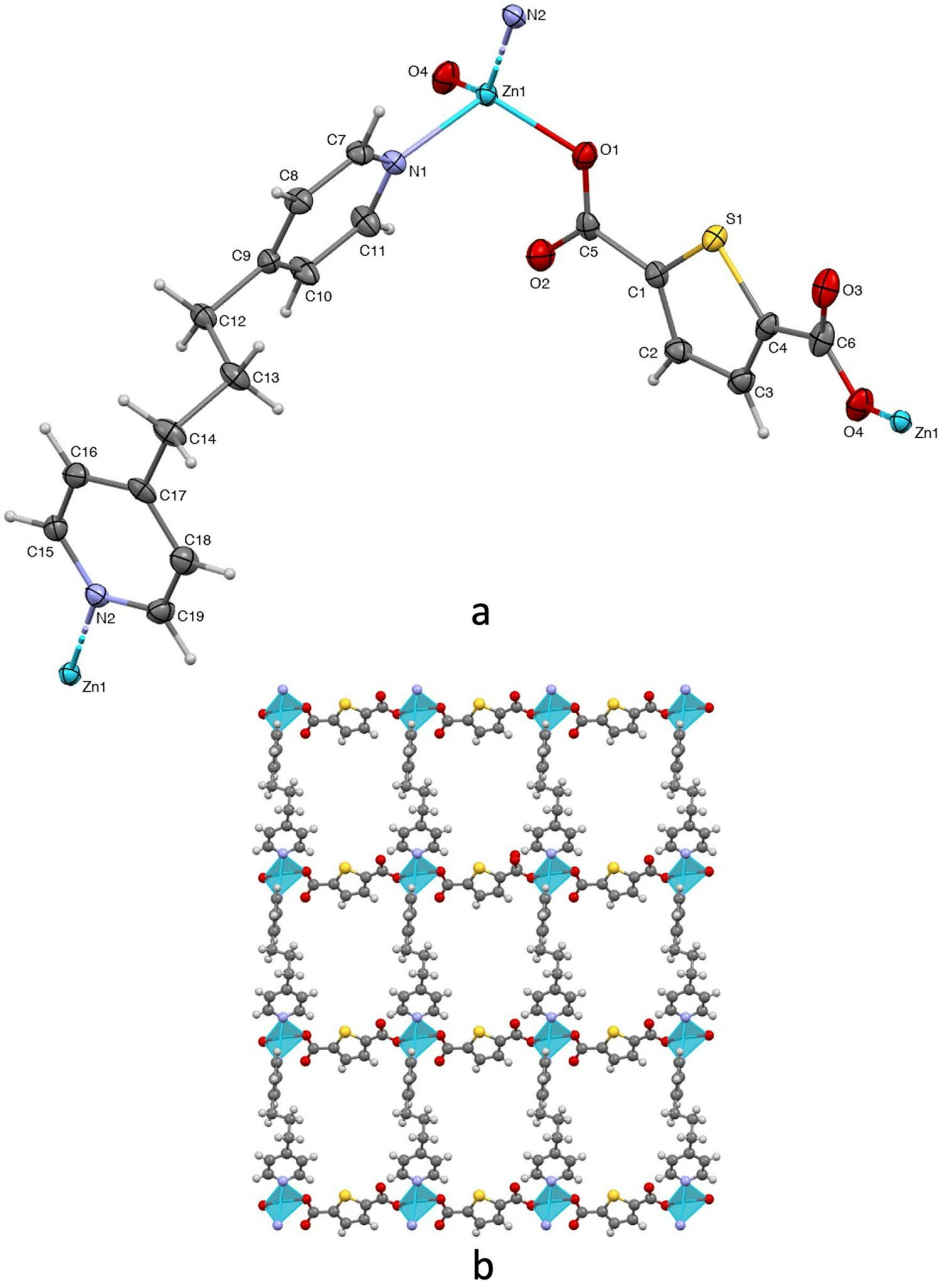
Molecular structure of **2** (ellipsoids shown at 60% probability) (**a**). 2D assembly of **2**; view down the *c* axis (**b**)

Polymer **2** crystallizes in the monoclinic space group P21/c. The asymmetric unit consists of one Zn(II), one tdc ligand and one tmb ligand. The four-coordinated Zn(II) is surrounded by two oxygens from two tdc ligands and two nitrogens from two tmb ligands, exhibiting a distorted tetrahedral coordination geometry (Fig. 2a). The Zn–O and Zn-N distances and, O–Zn–O, O-Zn-N and N–Zn–N bond angles ranging from 1.9342(11) Å to 2.0512(13) Å and from 96.87(5)° to 131.77(5)°, respectively, are analogous to those found in reported four-coordinated Zn(II) complexes [37, 38]. In polymer **2**, the tdc ligand, alike in polymer **1**, performs in a bridging monodentate coordination mode; meanwhile the tmb ligand joins with its characteristic bridging monodentate coordination mode. Consequently, polymer **2** displays a 2D coordination array with *sql* topology, as determined by the TOPOS-PRO software [39] analysis performed (Fig. 2b).

### Photoluminescence Properties

**Fig. 3:**
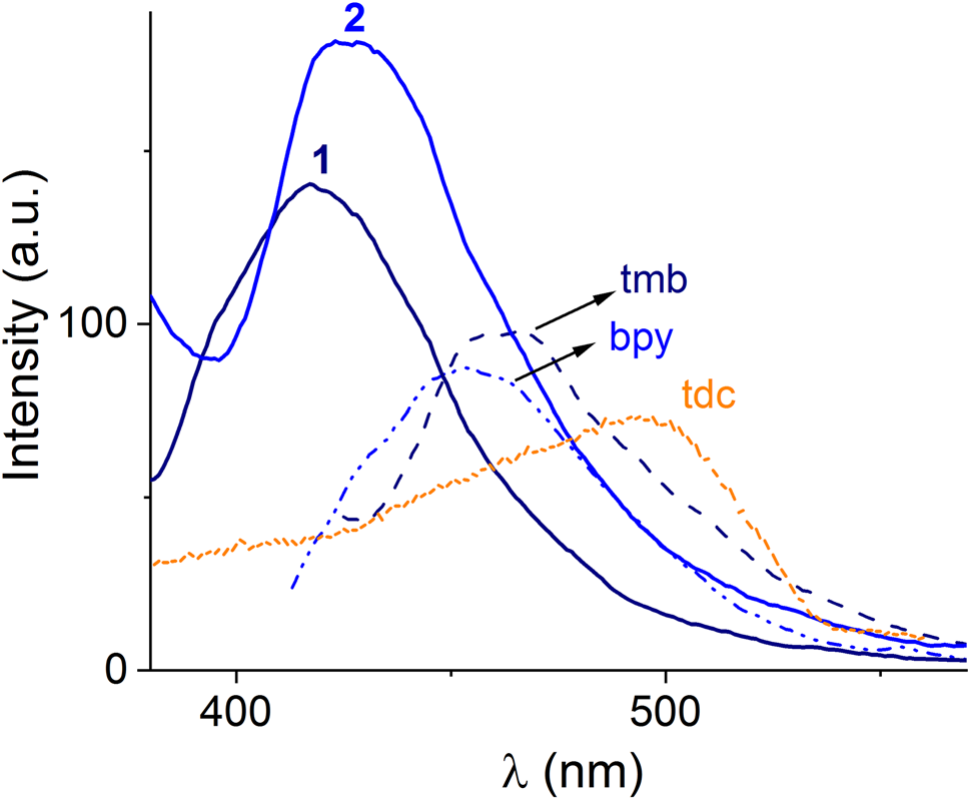
Solid-state photoluminescence spectra of **1** and **2** and free ligands tmb, bpy and tdc at r. t

As shown in Fig. 3, the solid-state photoluminescence spectra of crystalline samples of **1** and **2**, the free *N*-aromatic ligands (bpy and tmb) and H_2_tdc were measured under the same conditions (slit width = 5.0 nm).

The free ligands display broad emission bands centered at 454 nm (bipy, λ_ex_ = 340 nm), 466 nm (tmb, λ_ex_ = 340 nm) and 495 nm (tdc, λ_ex_ = 360 nm). These emission bands are typically assigned to π^*^ → n and π^*^ → π electronic transitions [20].

On the other hand, upon excitation at 330 nm, polymers **1** and **2** exhibit notable enhanced blue emission peaks at 418 nm for **1** and 426 nm for **2** in comparison to intensities observed for free ligands.

These blue shifts (ca. 35 nm) of **1** and **2** compared to those observed maxima for the free *N*-aromatic ligands can be assigned to a combination of ligand-centered electronic transitions (IL) [10] modified by the coordination of the aromatic ligand to the Zn(II) ions [40]. Furthermore, the improvement in intensity emission in this kind of Zn-polymers is related to 1) ligand-to-ligand charge transfers (LLCT) between the *N*-aromatic ligands that are constricted by coordination to metal atoms and 2) the increased rigidity of ligands inside the crystal arrangement, which reduces the loss of energy via non-radiative relaxation mechanisms [41].

The excitation spectra of **1** and **2** have bands ranging from 280 to 396 nm with maxima at λ_ex_ = 299 nm for **1** (λ_em_ = 410 nm) and λ_ex_ = 292 nm for **2** (λ_em_ = 410 nm) as displayed in Figures S4-S5.

Similar photoluminescence properties with maxima in the range 380–450 nm have been described to Zn-polymers containing *N*-aromatic donors and tdc such as [Zn(2,2´-dmbpy)(tdc)]_n_, [Zn_2_(3,3´-dmbpy)(tdc)_2_]_n_ [16], {[Zn(tdc)(bpfh)]·H_2_O}_n_ [17], [Zn_2_(tdc)_2_(bib)_2_]_n_(H_2_O) [15], and {[Zn_2_(bbmb)_2_(tdc)_2_]·H_2_O}_n_ [42] (2,2´-dmbpy = 2,2´-dimethyl-4,4´-bipyridine; 3,3´-dmbpy = 2,2´-dimethyl-4,4´-bipyridine; bpfh = bis(pyridylformyl) piperazine, bib = 1, 4-bis(imidazolyl)butane and bbmb = 1,4-bis(benzimidazol-1-yl-methyl)).

The quantum yields of aqueous ethanolic dispersion of **1**–**2** (Φ_**1**_ = 0.06, Φ_**2**_ = 0.09) were increased, compared to that of free bpy ligands (Φ_bpy_ = 0.03 and Φ_tmb_ = 0.04) which is consistent with the LLCT [5].

The higher emission intensity of polymer **2** compared to **1** can be induced by the absence of coordinated water molecules in **2**, which are efficient quenchers [43].

Considering that luminescent Zn-polymers **1**–**2** have multiple coordinating *O*- and *S*- atoms, we test their ability to sense divalent metal ions in solution, with the baseline expectation that divalent heavy atoms with environmental and chemical relevance such as Pb^2+^ and Hg^2+^ can be detected by direct coordination with the *S*- atoms of the tdc ligand, as has been successfully demonstrated in some organic polymers appended thiophene and benzothiazole rings [44, 45].

In the environmental context, Pb pollution is a persisting problem to human health, plants and animals, as well as a long danger to the environment. Even very low levels of Pb exposure can cause neurological cardiovascular and metabolic diseases. Thus, it is really important to develop a simple, inexpensive and rapid method for sensing Pb2 + with high sensitivity and selectivity [44, 45].

Zn-polymers **1–2** can be dispersed in 20% aqueous ethanol in the micromolar concentration range (< 50 μM) with chemical stability for several days, which was verified from the photoluminescence spectra corresponding to the starting aqueous ethanolic dispersion and after 48 h. In general, the spectra do not show evident changes during this time interval, indicating good stability in the medium. Therefore, these conditions were used for further spectroscopic studies. The dispersions of **1**–**2** are blue-emitting with maxima at 405 and 410 nm, respectively.

Next, cation sensing was carried out by steady state fluorescence spectroscopy. The luminescence responses of 20% aqueous ethanol dispersions of **1** and** 2** towards a series of nitrate salts of Pb^2+^, Hg^2+^, Cu^2+^, Cd^2+^, Ca^2+^, Mg^2+^, Mn^2+^, Fe^2+^, Co^2+^, Ni^2+^, Cu^2+^ and Zn^2+^ are shown in Figures S4 and 4, respectively.

**Fig. 4:**
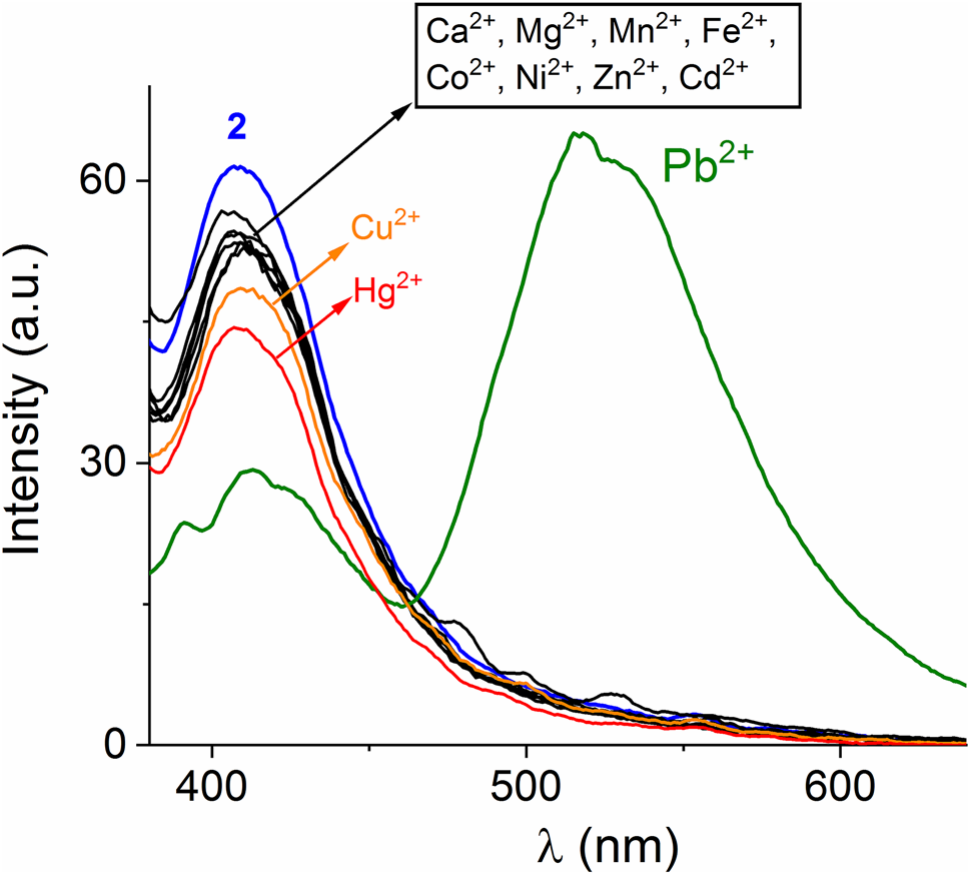
Luminescence spectra (λ_ex_ = 330 nm) of ethanol–water (v/v, 8/2) dispersion of **2** (10 μM) upon addition of different metal ions as nitrate salts ([M^2+^]_final_ = 40 μM)

For polymer **2,** the addition of Ca^2+^, Mg^2+^, Mn^2+^, Fe^2+^, Co^2+^, Ni^2+^, Cd^2+^ and Zn^2+^ gave a very low quenching effect *I*_F_ < 10% of its initial intensity *I*_o_ at 410 nm, as it is shown in Fig. 4. The addition of Cu^2+^ or Hg^2+^ reduces the photoluminescence starting intensity by about 20%.

Polymer **2** displayed the greatest change in emission towards Pb^2+^ exhibiting a quenching response of about 65% of the band at 410 nm, simultaneously, the appearance of a new emission broadband at 518 nm was clearly observed. A similar behavior induced by Pb^2+^, but with smaller changes in intensity, was observed in polymer **1** (Fig. S6).

**Fig. 5:**
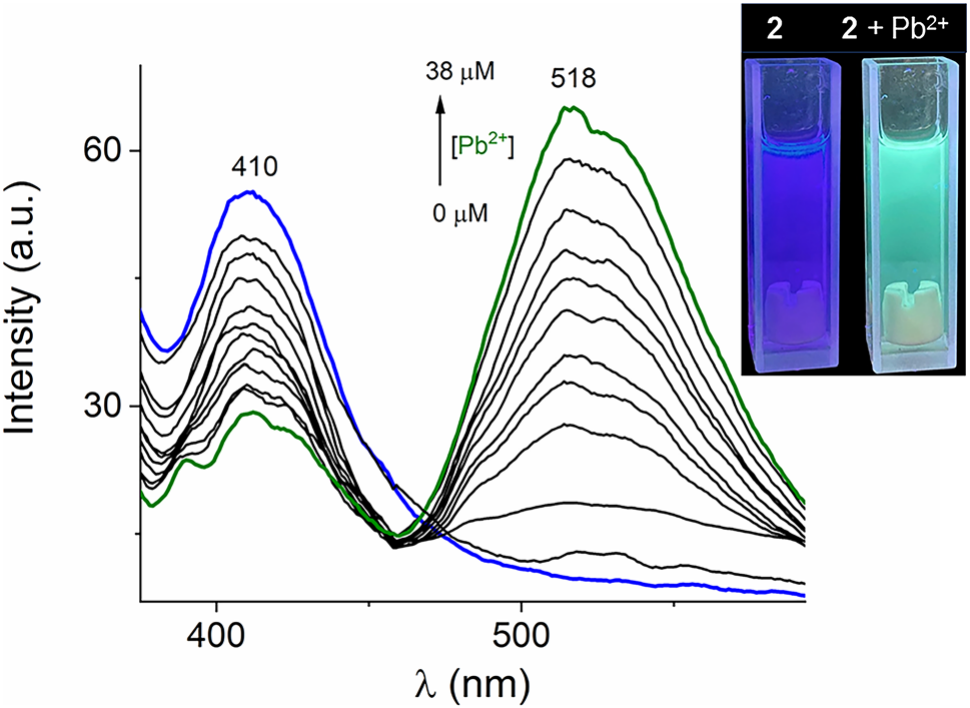
Fluorimetric titration (λ_ex_ = 330 nm) of **2** (10 μM) dispersed in ethanol–water (v/v, 8/2) upon addition of increasing amounts of Pb^2+^ (0–38 μM). Inset: luminescent response of dispersion of **2** upon addition of Pb^2+^ under UV light (365 nm)

To further insight into the chemosensing ability of polymer **2** towards Pb^2+^, a luminescent titration experiment was carried out under the same conditions, as shown in Fig. 5. As the concentration of Pb^2+^ (0–38 μM) increased the emission at 410 nm decreased, while a new concomitant strong emission band at 518 nm increased.

The *I*_518_/*I*_410_ ratio, within the micromolar concentration of Pb^2+^, is practically linear, so the optical change can be analyzed with a ratiometric treatment.

**Fig. 6:**
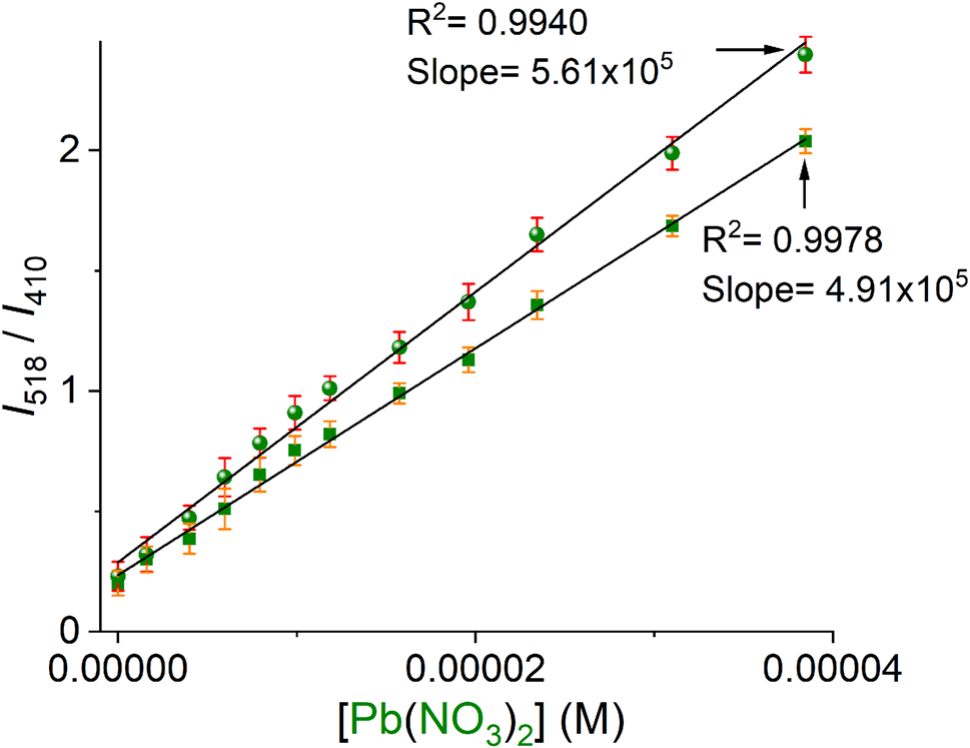
Ratiometric luminescent plot of **2** upon addition of Pb^2+^ ions without (●) and with (■) the presence of other metal ions ([M^2+^] = 100 mM each one; Ca^2+^, Mg^2+^, Mn^2+^, Fe^2+^, Co^2+^, Ni^2+^, Cu^2+^, Zn^2+^ and Hg^2+^) in 20% aqueous ethanol. Average of triplicate measurements

In the context of quantitative chemosensing, a ratiometric fluorescence analytical response is highly desired due to that the dual-emission fluorescent change can avoid errors caused by sensor concentration, environmental effects, and instrumentation [46]. Fig. 6 shows the ratio of the photoluminescent intensity measured at 518 and 410 nm (*I*_518_/*I*_410_) *versus* the increasing amount of Pb(NO_3_)_2_ in the absence and presence of common interfering metal ions (Ca^2+^, Mg^2+^, Mn^2+^, Fe^2+^, Co^2+^, Ni^2+^, Cu^2+^ and Zn^2+^). Notably, there was a linear dependence of the ratiometric response on the Pb^2+^ concentration in the range of 0–38 μM for both cases. The shape and slopes (*S*) of the ratiometric plots are very similar with the addition of up to ~ 4.0 equiv. of Pb^2+^, [Pb^2+^]_tot_ = 38 μM, indicating that this Zn-polymer **2** can operate as a chemosensor without any significant interference from other common environmental metal ions such as Cu^2+^, Zn^2+^, Ca^2+^, Mg^2+^ and Fe^2+^ even Hg^2+^. The discrimination between Pb^+2^ and Hg^2+^ is a highlight of this polymer. Such selectivity is still rare in literature. To gain further insight into the selectivity, we estimated an *apparent* binding constant between compound **2** and Pb^2+^ from the fluorimetric titration experiment (Fig. 5). Figure S7 shows the fluorimetric profile curve at 518 nm can be well fit to a 1:1 binding model by a nonlinear least-squares treatment to give an apparent binding constant of *K*_*(2:Pb)*_^=^ 5.62(± 0.06) × 10^4^ M^−1^. Such a value of the apparent affinity constant is similar to the best Pb sensors reported recently which ranging from 3.1 × 10^2^ M^−1^ to 9.5 × 10^7^ M^−1^ [47].

Next, the detection limit (LOD) of **2** towards Pb^2+^ was calculated by the Eq. 3*σ*/*S* [48], where *σ* is the standard deviation of photoluminescent intensity of blank for ten measurements and *S* is the slope of the ratiometric plot. LOD was estimated to be 1.78 ± 10 μM.

The literature features very few examples of luminescent chemosensors selective to Pb^2+^ over Hg^2+^ and other heavy metal ions based on transition/lanthanide-metal coordination complexes [30, 32, 49, 50].

Some recent examples of luminescent sensors for the detection and quantification of Pb^2+^ in the aqueous media are compiled in Table 1.

**Table 1:** A comparison of luminescent chemosensors for the detection of Pb^2+^ ions

Pb^2+^ ions chemosensors	Analytical signal	Media	LOD	Ref
carboxyl-based Zn-MOF	turn-off	H_2_O	8.0 μM	[30]
tetrazol-isophthalate-based Zn-MOF	turn-on	H_2_O	10 μM	[32]
fluorene-based La-MOF	turn-off	H_2_O	8.2 μM	[49]
3,5-dicarboxyphenol-based Tb-MOF	turn-off	H_2_O	100 nM	[50]
hexakis(phenylthio)benzene compound	turn-on	H_2_O	6.0 μM	[51]
dichlorosalicylaldehyde-based half-salamo	turn-on	DMF-H_2_O	56 nM	[47]
thiosemicarbazide-naphthalimide derivative	turn-on	CH_3_CN-H_2_O	4.7 nM	[52]
rhodamine 6G derivative	turn-on	CH_3_CN-H_2_O	27 nM	[53]
ethanethiol-based bis-Schiff-base	turn-on	CH_3_CN/H_2_O	380 nM	[54]
thiophenedicarboxylate-based Zn-CP, **2**	ratiometric	EtOH/H_2_O	1.7 μM	This work

As can be seen from the analytical signals in Table 1, our system, compound **2**, is the first example of a ratiometric fluorescent sensor for Pb^2+^ ions.

### Recognition Mechanism Investigation

The sensing mechanism of **2** towards Pb^2+^ ions was investigated by ^1^H NMR spectroscopy, ATR-IR measurements, and scanning electron microscopy with energy-dispersive X-ray spectroscopy (SEM–EDS). The treatment of crystals of **2** (30 mg) with 5.0 equiv. of Pb(NO_3_)_2_ in 20 mL of ethanol–water (v/v, 8/2) abruptly produced a gray amorphous precipitate. This solid was filtered off, washed with ethanol–water and vacuum dried at r.t.

**Fig. 7:**
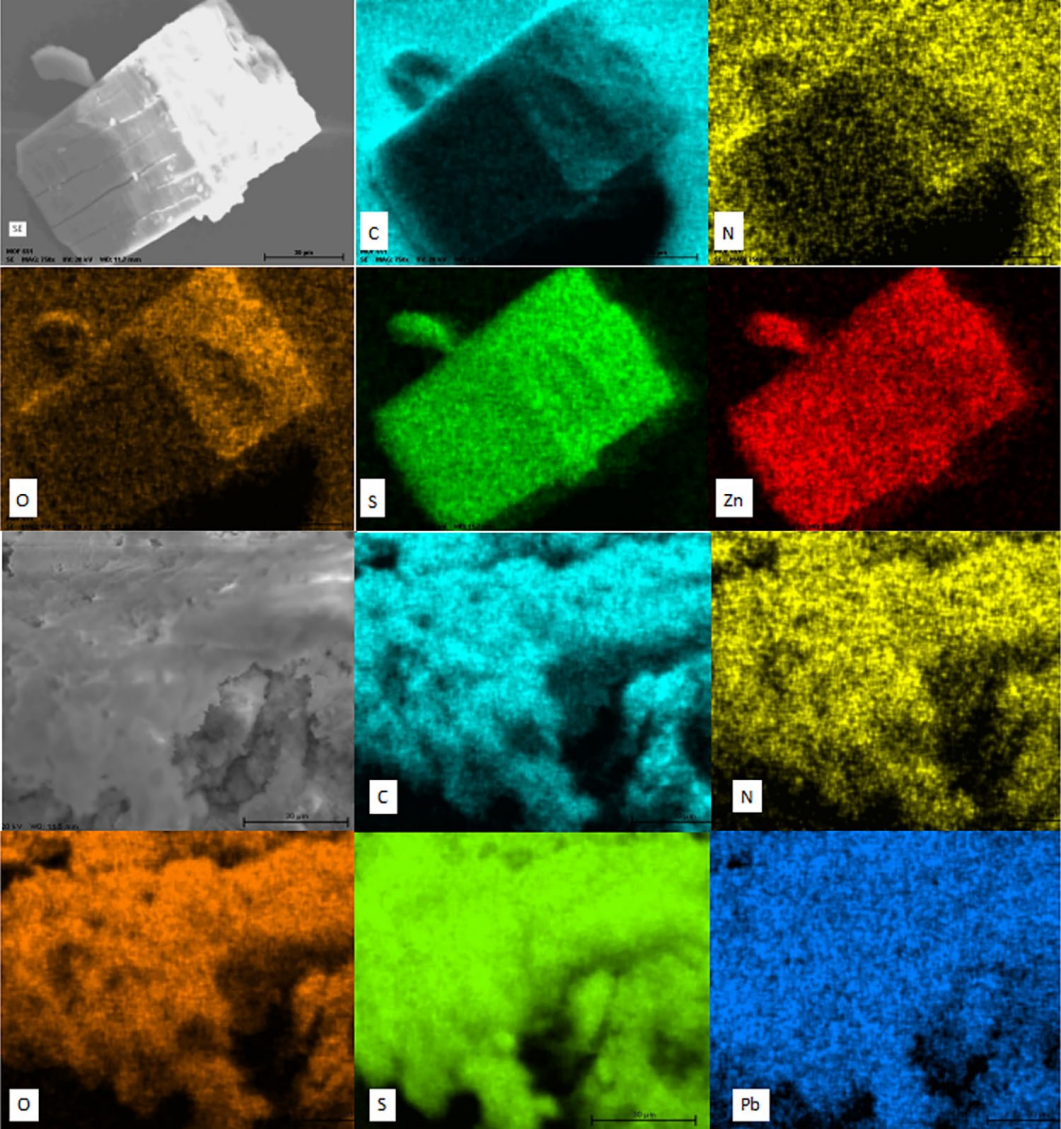
SEM–EDS mapping images of the as-synthesized **2** (top images) and of **2** after contact with Pb^2+^ (bottom images)

Next, the starting crystals of **2** and the solid from the Pb^2+^ treatment were analyzed by SEM–EDS. SEM image of** 2** is shown in Fig. 7 (top image), a crystalline material with well-defined facets and edges can be observed. Figure 7 also shows the EDS elemental chemical mapping of **2** before and after contact with Pb^2+^, respectively. C, N, O, S and Zn signs with homogeneous distributions are observed in the as-synthesized single-crystals of **2** (Fig. 7, top images).

After treatment of **2** with Pb^2+^ there is an evident change in the crystalline morphology of **2** to a coarse material formed by layers and agglomerates and, surprisingly, there are not Zn signals, appearing instead the Pb signs (Fig. 7, bottom images). The SEM–EDS spectra and tables with the analyses results are shown in Figure S8 of the ESI.

**Fig. 8:**
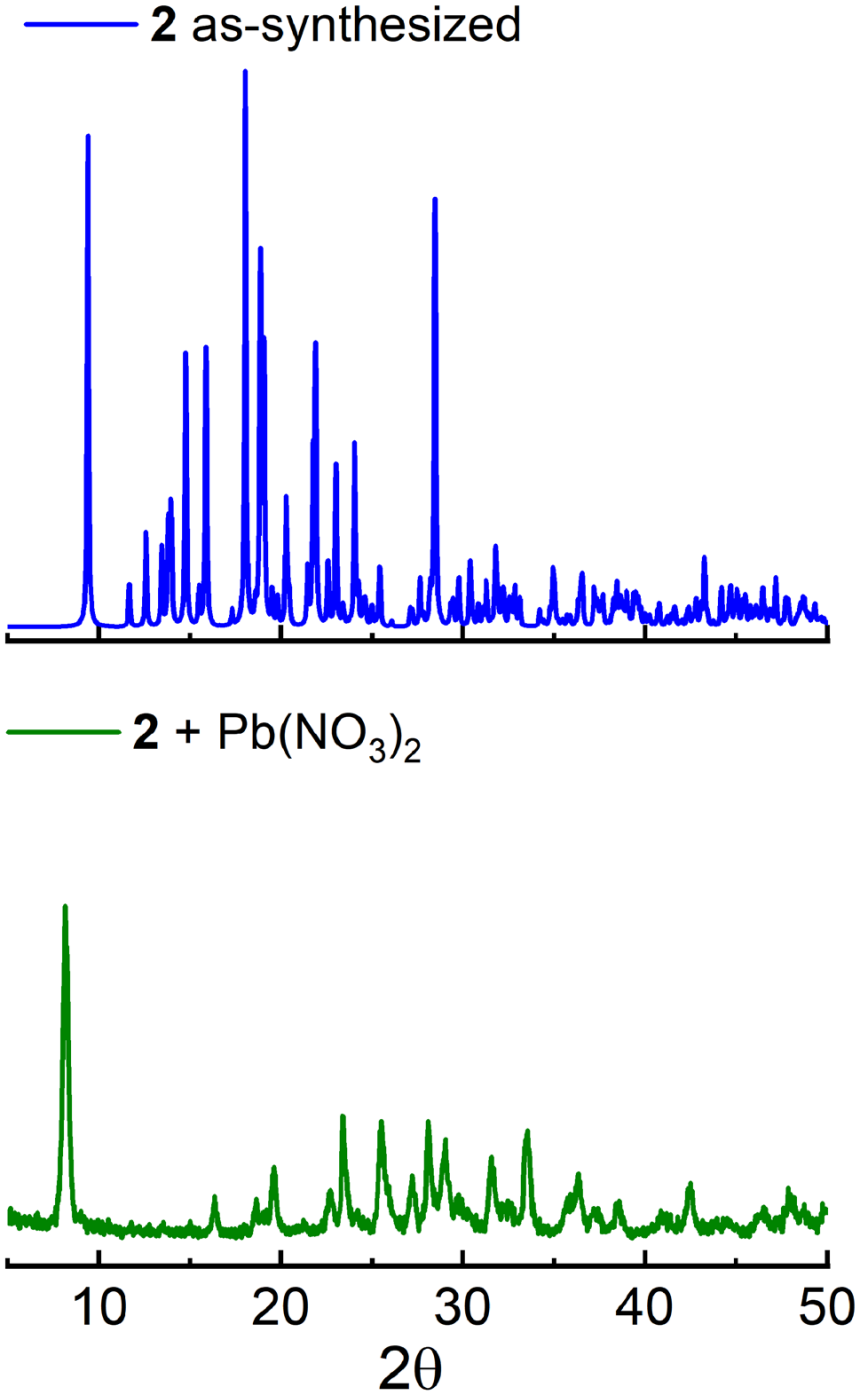
Powder X-ray diffraction patterns of a crystalline sample of** 2** and **2** treated with Pb^2+^ in ethanol–water

These results strongly suggest an exchange of metal ions from Zn^2+^ for Pb^2+^ which may explain the appearance of the new green emission band at 518 nm. Reports in the context of mesoporous materials have displayed that thiophene-based polymers can perform heavy metal ion adsorption with high affinity towards Hg^2+^ or Pb^2+^ ions [55]. Next, PXRD pattern of **2** and **2** treated with Pb(NO_3_)_2_ were recorded. The most intense peaks of the crystalline sample of **2** at 9.48° (0 0 1), 17.81° (1 2 -1), 18.87 (1 0 -3) and 28.44 (0 3 3) disappeared after treating it with Pb^2+^, in contrast, the resulting PXRD spectrum showed low intense and wide bands (Fig. 8) which indicates that the material is amorphous as observed in the SEM microscopy.

The precipitate obtained by treatment with Pb^+2^ is insoluble in DMSO, DMF, acetonitrile, alcohols, and non-polar solvents. Therefore, to verify the presence of the ligands (tmb and tdc) in the precipitate, this solid was dissolved in DMSO-*d*_6_ containing deuterium chloride (2 vol %, v/v).

**Fig. 9:**
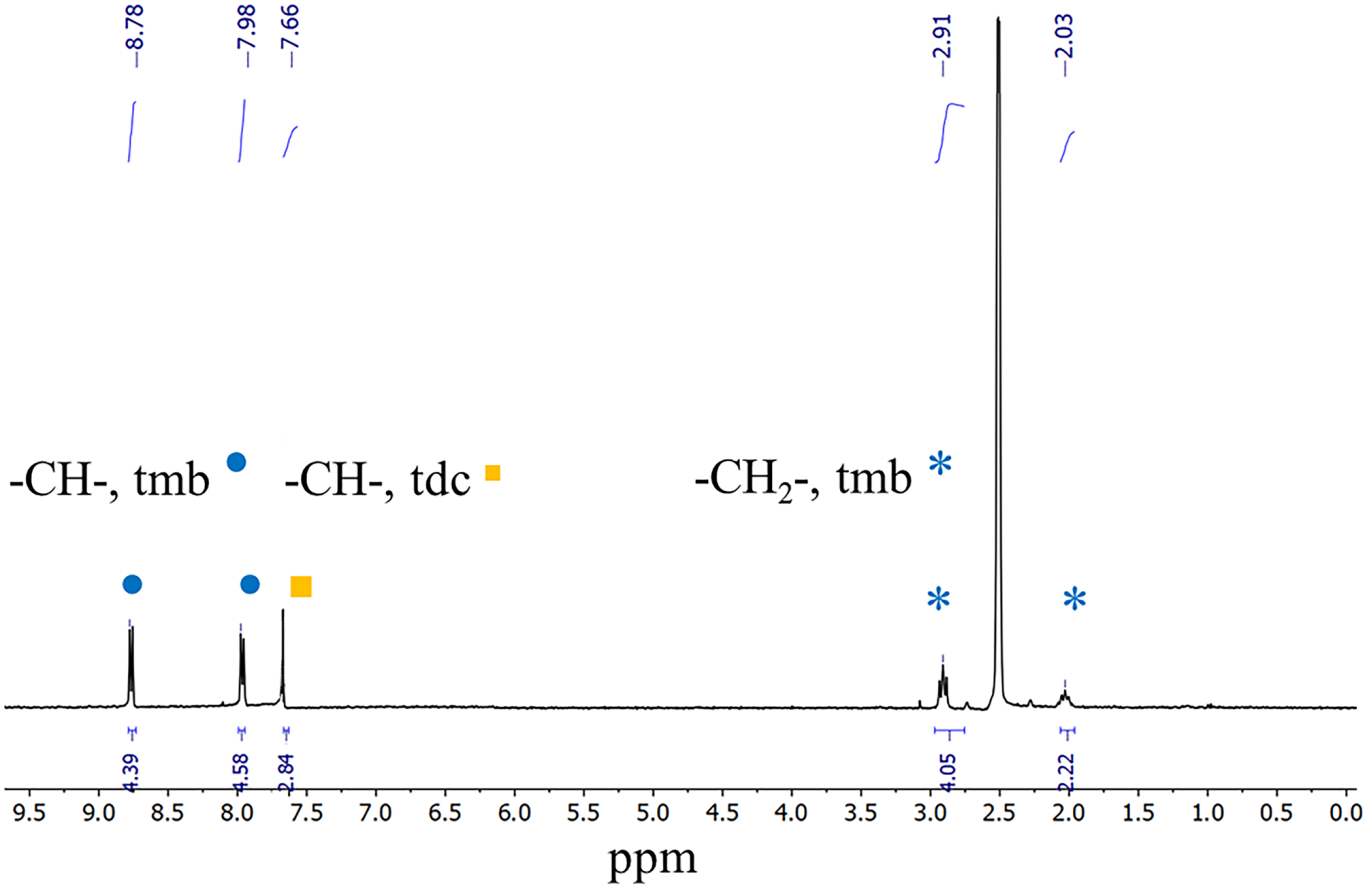
^1^H NMR (300 MHz 25 °C) spectrum of **2** treated with Pb^2+^ dissolved in DMSO-*d*_*6*_ containing 2% vol of DCl

^1^H NMR spectrum established the retention of tmb and tdc ligands as is shown in Fig. 9. The proton peak integration was consistent with a 1:1 ratio between the ligands.

**Fig. 10:**
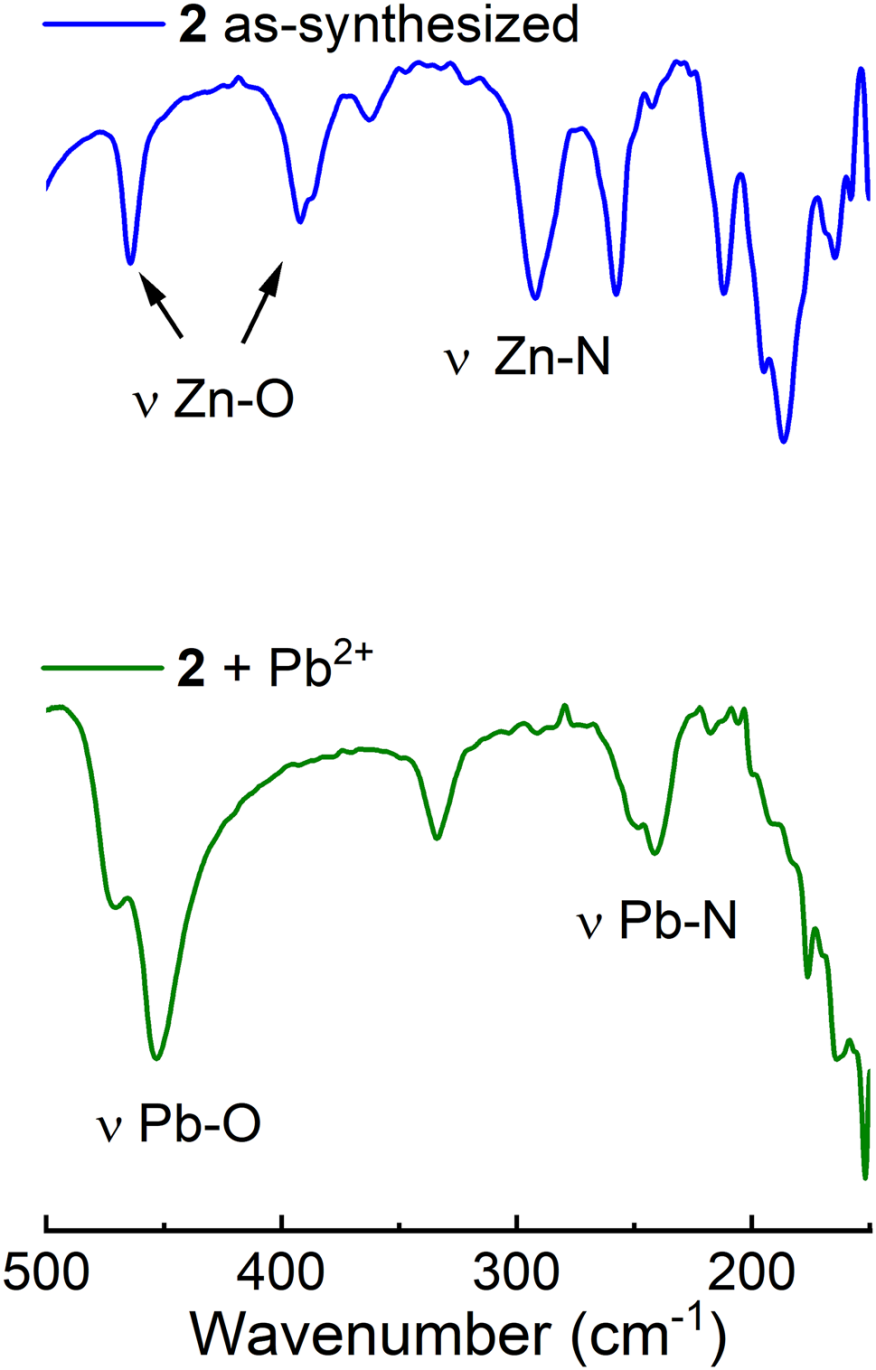
Far infrared spectra of **2** and **2** treated with Pb^2+^

A comparison of far infrared spectra of **2** and **2** treated with Pb^2+^ is shown in Fig. 10. The Pb–O and Pb-N stretching frequencies of **2** treated are clearly observed at 440 and 230 cm^−1^ [56, 57].

In contrast, the Zn-N (300 cm^−1^) and Zn–O (400 cm^−1^) stretching frequencies observed in Zn-polymer **2** practically disappear after treating it with Pb^2+^.

Moreover, the initial liquid phase from the reaction mixture of **2** with Pb^2+^ was dried under vacuum and analyzed by ATR-IR (Fig. S9). The spectrum showed the characteristic bands for Zn(NO_3_)_2_ hydrated at 830 cm^−1^ and 1390 cm^−1^ assigned to vibrational modes of the NO_3_^−^ ion as shown in Figure S8; therefore, this salt is a by-product of the metal-exchange reaction.

The efficient metal-exchange process of Zn-compound with Pb may be driven by the high affinity of Pb for *S*- and *O*- atoms, bond dissociation energies of Pb–S, 398 kJmol^−1^ and Pb–O, 382 kJmol^−1^ [58].

## Conclusions

Two novel flexible Zn(II) coordination polymers **1** and** 2** have been synthesized by self-assembly reactions combining thiophenedicarboxylate and bipyridines in aqueous methanol. X-ray crystallography studies revealed 1D and 2D arrays, respectively. Photoluminescence properties of **1**–**2** in the solid state and suspensions in aqueous ethanol show that they possess blue emission with maxima at 410 nm.

The Zn-thiophene polymer **2** can be used as a luminescent chemosensor towards Pb^2+^ ions in the micromolar concentration range in neutral aqueous ethanol with high selectivity over other common heavy metal ions including Fe^2+^, Zn^2+^, Cu^2+^, Hg^2+^ and Cd^2+^, which are common interferences. Under these conditions, the addition of Pb^2+^ to the dispersion of **2** exhibits a fast fluorescent ratiometric response with a detection limit of 1.78 ± 10 μM.

The luminescent sensing mechanism can be attributed to a metal-ion exchange reaction with the formation of a new Pb-complex with green emission at 518 nm.

Considering the foregoing SEM–EDS results and spectroscopic evidence, the luminescent sensing mechanism of Pb^2+^ by the Zn-polymer **2** can be assigned to an efficient metal-exchange reaction with the simultaneous release of the Zn(NO_3_)_2_ salt as a byproduct.

Overall, these results further highlight the utility of low-cost and simple luminescent Zn-coordination polymers for highly toxic and pollutant cation sensing in aqueous phase.

## Supplementary Information

Below is the link to the electronic supplementary material.

Supplementary file1 (PDF 898 kb)

## Data Availability

No datasets were generated or analysed during the current study.
