# Supplementary material for: Highly Selective Ratiometric Sensors for Pb2+ Based on Luminescent Zn(II)-Coordination Polymers with Thiophenedicarboxylate. Crystal Structures and Spectroscopic Studies
Source: J Fluoresc. 2024 May 28;35(5):3449–59. doi: 10.1007/s10895-024-03754-1 (PMC12095416; doi:10.1007/s10895-024-03754-1)
Supplement: Supplementary file 1 — Supplementary file1 (PDF 898 kb) [file 10895_2024_3754_MOESM1_ESM.pdf]

Highly Selective Ratiometric Sensors for Pb^{2+} based on Luminescent Zn(II)-Coordination Polymers with Thiophenedicarboxylate. Crystal Structures and Spectroscopic Studies

Georgina M. Otero-Fuentes,[a] Victor Sánchez-Mendieta,[b]* Alejandro Sánchez-Ruiz,[c] Raúl A. Morales-Luckie,[b] Diego Martínez-Otero,[b] Jonathan Jaramillo García,[d] Juan Pablo León-Gómez[c] and Alejandro Dorazco-González[c]*

[a] *Facultad de Química, Universidad Autónoma del Estado de México. Paseo Colón y Paseo Tollocan. Toluca, Estado de México, 50120, México.*
[b] *Centro Conjunto de Investigación en Química Sustentable UAEM-UNAM. Carretera Toluca -Atlacomulco Km. 14.5, San Cayetano, Toluca, Estado de México, 50200, México. E-mail: vsanchezm@uaemex.mx*
[c] *Instituto de Química, Universidad Nacional Autónoma de México. Circuito Exterior, Ciudad Universitaria, Ciudad de México, 04510, México. E-mail: adg@unam.mx*
[d] *Tecnológico Nacional de México, Campus Zitácuaro (ITZ), Av. Tecnológico 186, Colonia Manzanillos, 61534, H. Zitácuaro, Michoacán, México*

Additional Information

Table S1. Crystal data and structure refinement parameters for **1** and **2**.

Table S2. Selected bond distances (Å), angles (˚) and hydrogen bonding for **1**.

Table S3. Selected bond distances (Å), angles (˚) and hydrogen bonding for **2**.

Figure S1. FTIR-ATR spectrum of polymer **1**.

Figure S2. FTIR-ATR spectrum of polymer **2**.

Figure S3. Supramolecular structure of polymer **1** showing the 3D array.

Figure S4. Solid-state excitation (black) and emission (navy) spectra of **1** at room temperature.

Figure S5. Solid-state excitation (black) and emission (blue) spectra of **2** at room temperature

Figure S6. Luminescence spectra of ethanol-water dispersion of **1** upon additions of different metal ions.

Figure S7. Fluorimetric titration curve of **2** upon addition of increasing amounts of Pb^{2+}.

Figure S8. SEM-EDS spectra and tables for polymer **2** and polymer **2** treated with Pb^{2+}.

Figure S9. FTIR-ATR spectra of mother liquor of reaction between polymer **2** and $Pb(NO_3)_2$ (top) and the $Zn(NO_3).6H_2O$ salt.

Table S1. Crystal data and structure refinement parameters for **1** and **2**.

Empirical formula	$C_{22}H_{22}N_2O_{13}S_2Zn_2$ (**1**)	$C_{19}H_{16}N_2O_4SZn$ (**2**)
Formula weight	717.27	433.77
Temperature (K)	100(2)	
Wavelength (Å)	0.71073	
Crystal system	Triclinic	Monoclinic
Space group	*P-1*	*P2₁/c*
a (Å)	6.8834(3)	9.8821(2)
b (Å)	9.6666(4)	11.9911(3)
c (Å)	10.5163(4)	15.2396(3)
α (°)	83.7360(7)	90
β (°)	81.3253(7)	90.1024(9)
γ (°)	71.4635(7)	90
Volume ($Å^3$)	654.46(5)	1795.62(7)
Z	1	4
D_{calc} (Mg/m^3)	1.820	1.605
Absorption coefficient (mm^{-1})	2.065	1.513
F(000)	364	888
Crystal size (mm^3)	0.228 x 0.206 x 0.170	0.388 x 0.356 x 0.286
Theta range for data collection (°)	1.963 to 27.512	2.073 to 27.442
Index ranges	-8<=h<=8, -12<=k<=12, -13<=l<=13	-12<=h<=12, -15<=k<=15, -18<=l<=19
Reflections collected	12547	34213
Independent reflections	3005 [R(int) = 0.0182]	4099 [R(int) = 0.0352]
Refinement method	Full-matrix least-squares on F^2	
Data/restraints/parameters	3005 / 36 / 226	4099 / 49 / 263
Goodness-of-fit on F^2	1.080	1.070
Final R indices [I>2sigma(I)]	R1 = 0.0192, wR2 = 0.0520	R1 = 0.0248, wR2 = 0.0607
R indices (all data)	R1 = 0.0203, wR2 = 0.0525	R1 = 0.0261, wR2 = 0.0612
Largest diff. peak and hole (e.$Å^{-3}$)	0.431 and -0.229	0.713 and -0.313

Table S2. Selected bond distances (Å), angles (˚) and hydrogen bonding for **1**.

Bond lengths (Å)			
Zn(1A)-O(1)	1.9385(16)	Zn(1)-O(1)	1.9788(16)
Zn(1A)-O(5A)	1.998(8)	Zn(1)-N(1)	2.0242(17)
Zn(1A)-O(4)#1	2.0121(16)	Zn(1)-O(5)	2.164(8)
Zn(1A)-N(1)	2.0794(17	Zn(1)-O(6)	2.179(2)
Zn(1)-O(4)#1	1.9084(16)		

Angles (˚)			
O(1)-Zn(1A)-O(5A)	107.9(2)	O(4)#1-Zn(1)-O(5)	92.9(3)
O(1)-Zn(1A)-O(4)#1	112.86(7)	O(1)-Zn(1)-O(5)	91.4(2)
O(1)-Zn(1A)-N(1)	95.11(7)	N(1)-Zn(1)-O(5)	92.9(3)
O(5A)-Zn(1A)-N(1)	104.3(3)	O(4)#1-Zn(1)-O(6)	83.60(7)
O(4)#1-Zn(1A)-N(1)	134.95(7)	O(1)-Zn(1)-O(6)	94.05(8)
O(4)#1-Zn(1)-O(1)	115.73(8)	N(1)-Zn(1)-O(6)	87.80(7)
O(4)#1-Zn(1)-N(1)	147.90(8)	O(5)-Zn(1)-O(6)	174.4(2)
O(1)-Zn(1)-N(1)	95.64(7)		

D-H...A	d(D-H)	d(H...A)	d(D...A)	<(DHA)
O(5A^b)-H(5A^b)...O(2)#4	0.95(3)	1.71(3)	2.655(11)	172(4)
O(5A^b)-H(5B^b)...O(2)#5	0.59(4)	2.24(4)	2.729(9)	141(5)
O(5^a)-H(5C^a)...O(2)#4	0.66(4)	2.09(4)	2.754(10)	174(5)
O(5^a)-H(5D^a)...O(2)#5	1.17(3)	1.74(3)	2.893(8)	169(3)
O(6^a)-H(6A^a)...O(6^a)#6	0.826(10)	1.94(2)	2.473(4)	121(2)
O(6^a)-H(6A^a)...O(7)#6	0.826(10)	2.093(12)	2.834(2)	149(2)
O(6^a)-H(6B^a)...O(7)	0.834(10)	1.890(17)	2.682(2)	158(3)
O(7)-H(7A)...O(4)#7	0.846(9)	2.073(10)	2.8506(14)	152.5(14)
O(7)-H(7B)...O(3)#8	0.839(9)	1.979(10)	2.8115(15)	170.9(19)

Symmetry transformations used to generate equivalent atoms:

#1 x-1,y+1,z #2 -x,-y+1,-z+1 #3 x+1,y-1,z #4 x-1,y,z #5 -x+1,-y+2,-z #6 -x+1,-y+2,-z+1

#7 -x+2,-y+1,-z+1 #8 -x+1,-y+1,-z+1

Table S3. Selected bond distances (Å), angles (˚) and hydrogen bonding for **2**.

Bond lengths (Å)			
Zn(1)-O(1)	1.9342(11)	Zn(1)-N(2)#2	2.0508(13)
Zn(1)-O(4)#1	1.9550(12)	Zn(1)-N(1)	2.0512(13)
**Angles (˚)**			
O(1)-Zn(1)-O(4)#1	114.36(5)	O(1)-Zn(1)-N(1)	109.70(5)
O(1)-Zn(1)-N(2)#2	96.87(5)	O(4)#1-Zn(1)-N(1)	97.61(5)
O(4)#1-Zn(1)-N(2)#2	131.77(5)	N(2)#2-Zn(1)-N(1)	105.55(5)

Symmetry transformations used to generate equivalent atoms:

#1 x-1,y,z #2 x,y-1,z #3 x,y+1,z #4 x+1,y,z

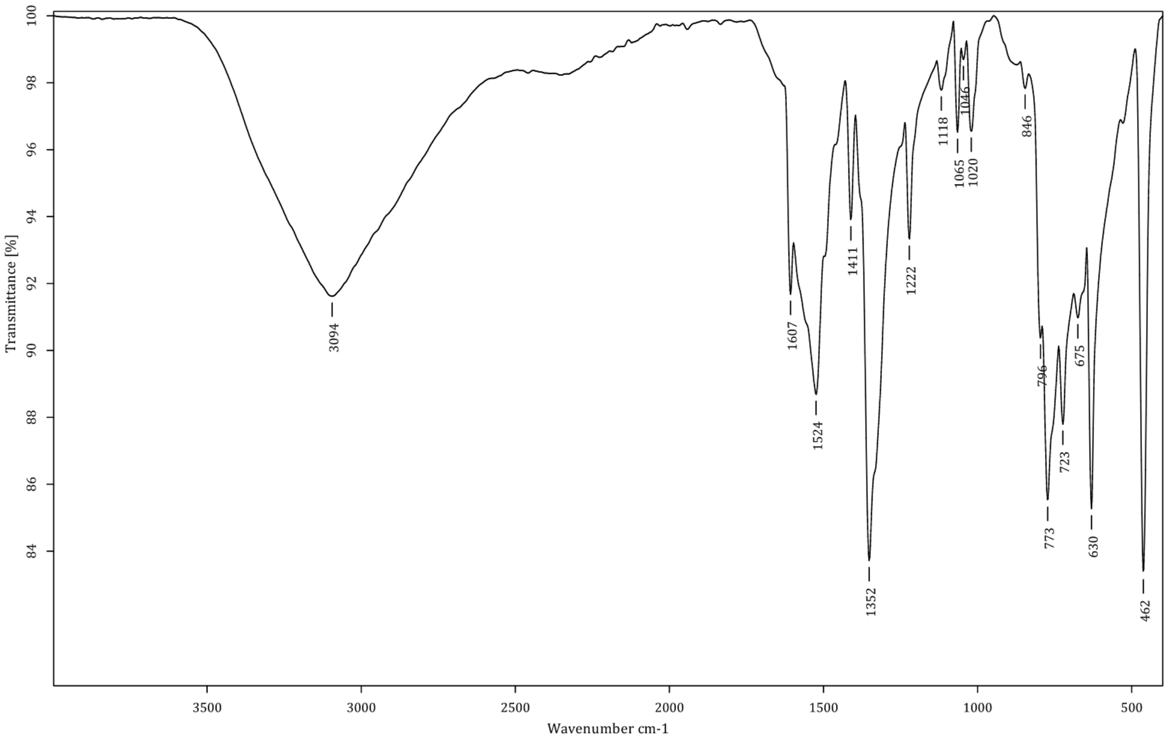

Figure S1. FTIR-ATR spectrum of polymer **1**.

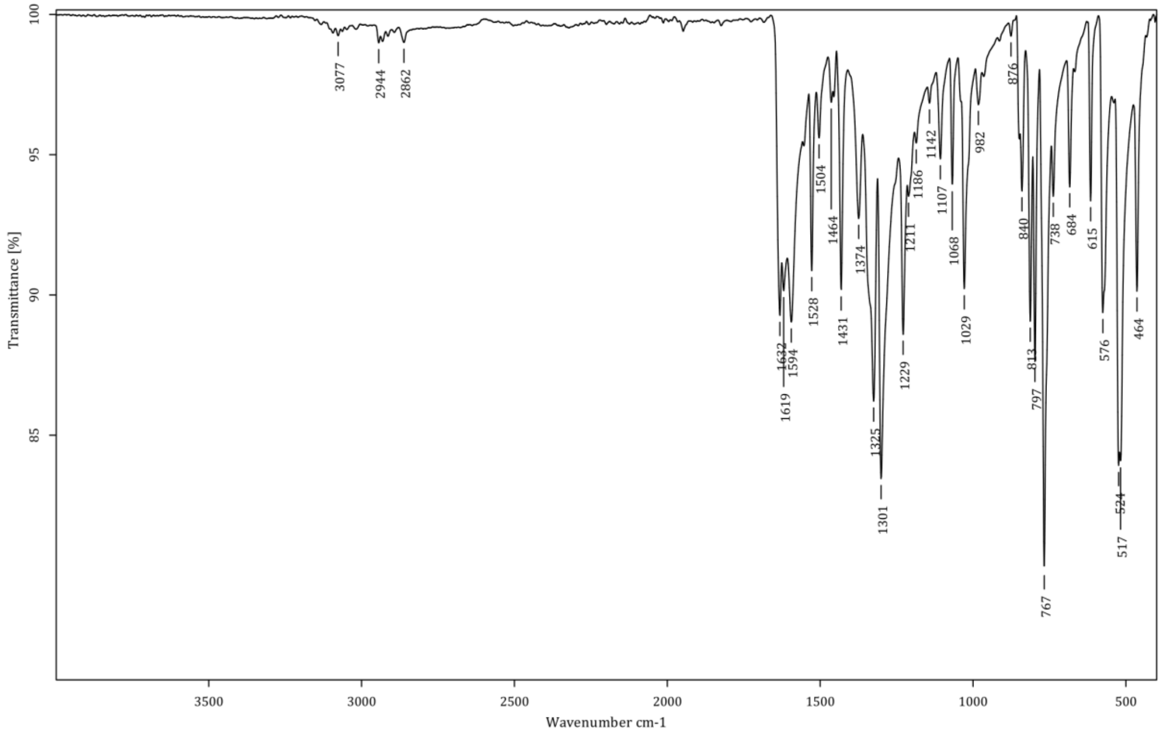

Figure S2. FTIR-ATR spectrum of polymer **2**.

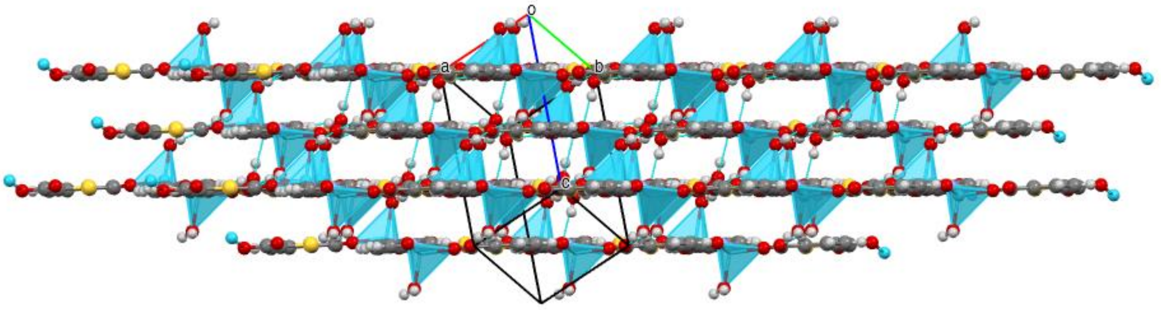

Figure S3. Supramolecular structure of polymer **1** showing the 3D array.

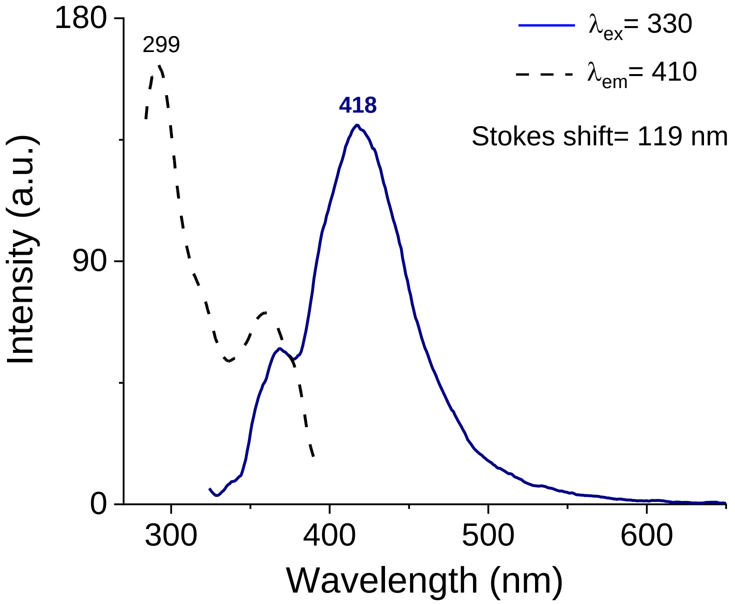

Figure S4. Solid-state excitation (black) and emission (navy) spectra of **1** at room temperature.

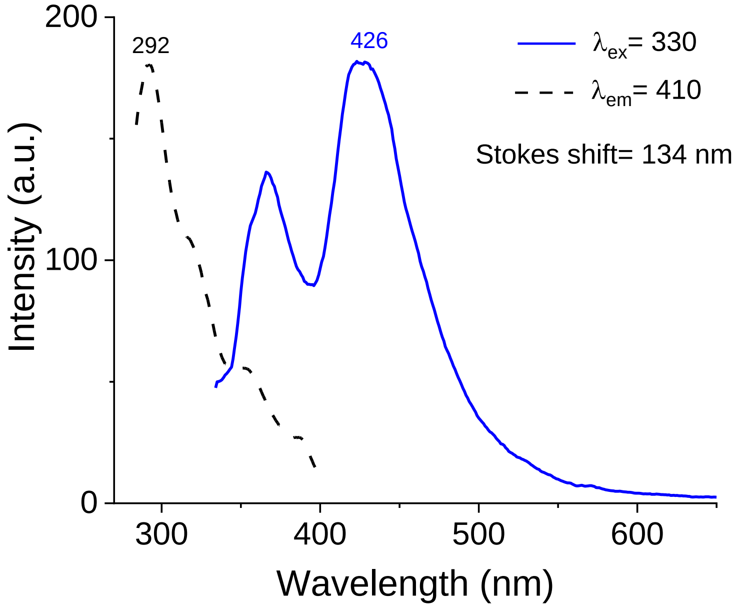

Figure S5. Solid-state excitation (black) and emission (blue) spectra of **2** at room temperature

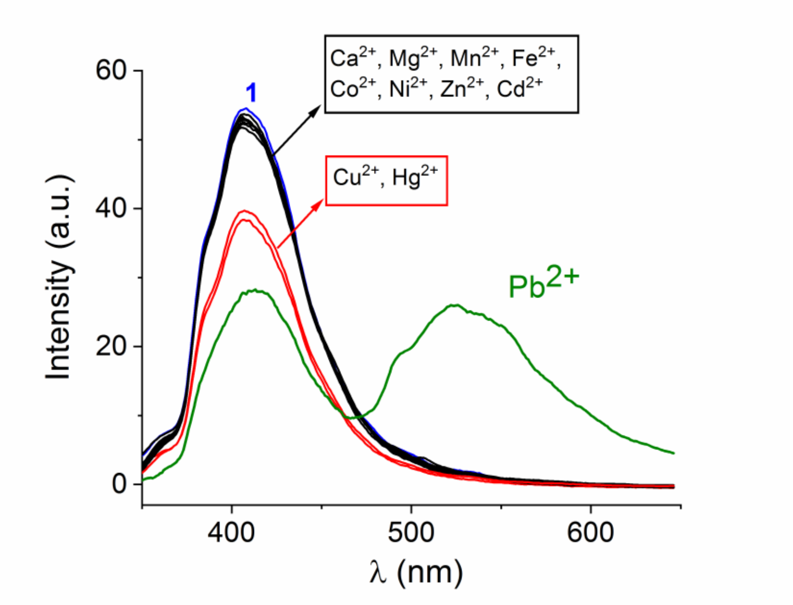

Figure S6. Luminescence spectra (λ_{ex}= 330 nm, 25°C) of ethanol-water (v/v, 8/2) dispersion of **1** (10 μM) upon additions of different metal ions as nitrate salts ($[M^{2+}]_{final}$= 50 μM).

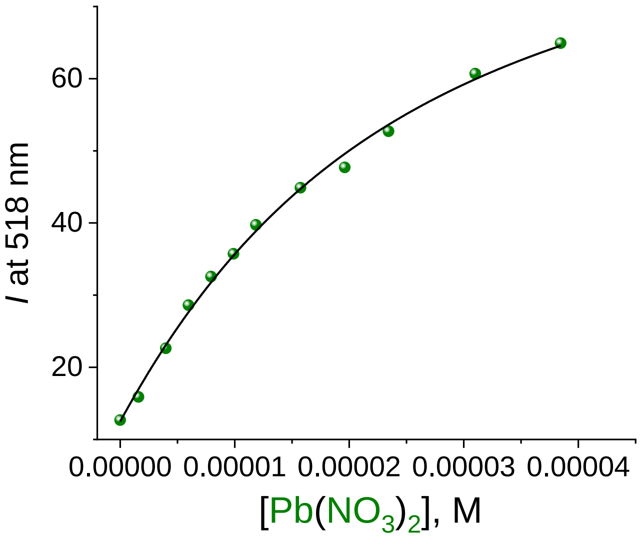

Figure S7. Fluorimetric titration curve (λ_{ex} = 330 nm) of **2** (10 μM) dispersed in ethanol–water (v/v, 8/2) upon addition of increasing amounts of Pb^{2+} (0-38 μM). The solid line was obtained by fitting of data to the theoretical equation to the 1:1 union model.

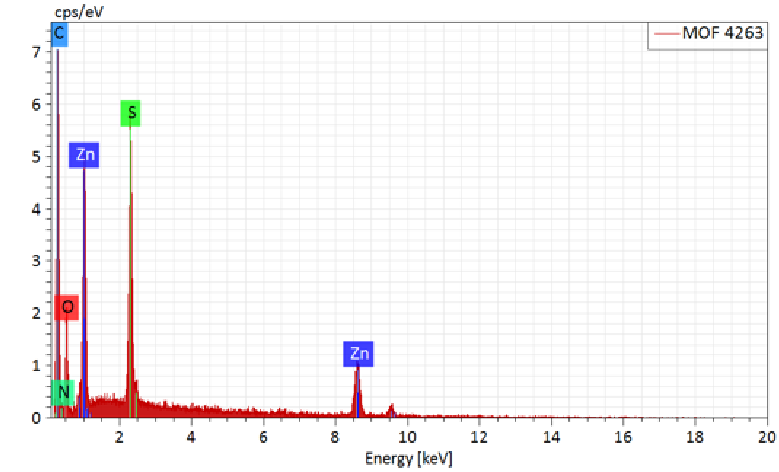

MOF 4263

Element	At. No.	Netto	Mass [%]	Mass Norm. [%]	Atom [%]	abs. error [%] (1 sigma)	rel. error [%] (1 sigma)
Zinc	30	2784	12.59	12.59	2.86	0.34	2.70
Carbon	6	8376	55.38	55.38	68.51	2.89	5.21
Nitrogen	7	474	9.60	9.60	10.19	0.54	5.65
Sulfur	16	7468	5.16	5.16	2.39	0.16	3.18
Oxygen	8	2168	17.27	17.27	16.04	0.93	5.38
		Sum	100.00	100.00	100.00		

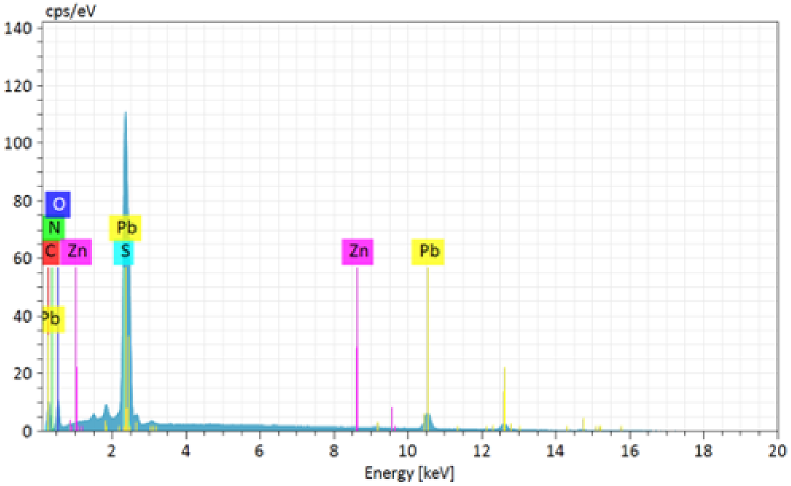

MOF 1 8458

Element	At. No.	Netto	Mass [%]	Mass Norm. [%]	Atom [%]	abs. error [%] (1 sigma)	rel. error [%] (1 sigma)
Carbon	6	4803	6.44	6.44	25.79	0.36	5.59
Oxygen	8	12612	15.82	15.82	47.61	0.84	5.33
Sulfur	16	27903	4.03	4.03	6.05	0.14	3.52
Nitrogen	7	475	1.07	1.07	3.67	0.08	7.60
Zinc	30	0	0.00	0.00	0.00	0.00	2.51
Lead	82	23008	72.65	72.65	16.88	1.84	2.53
		Sum	100.00	100.00	100.00		

Figure S8. SEM-EDS spectra and tables for polymer **2** (top spectra and table) and polymer **2** treated with Pb^{2+} (bottom spectra and table).

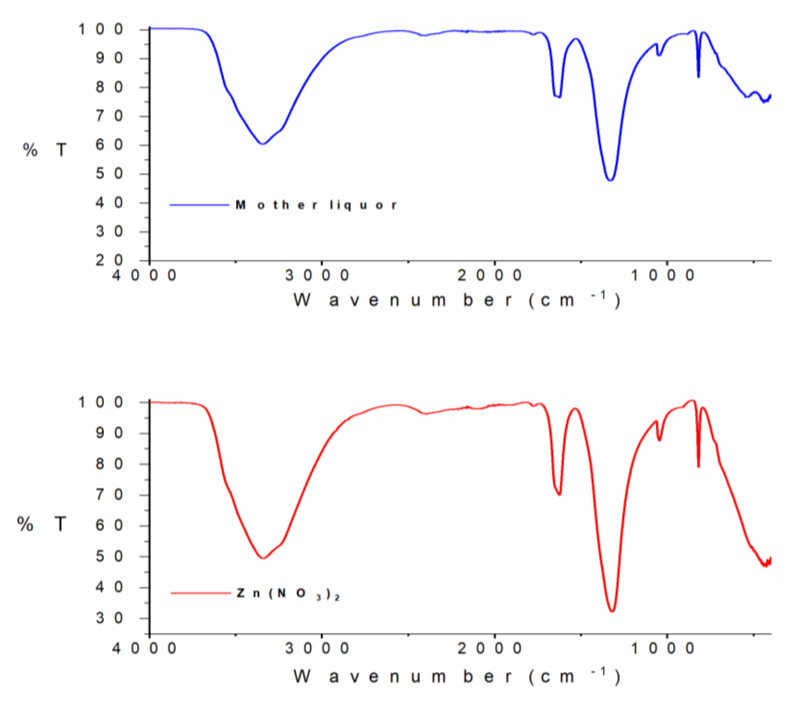

Figure S9. FTIR-ATR spectra of mother liquor of reaction between polymer **2** and $Pb(NO_3)_2$ (top) and the $Zn(NO_3).6H_2O$ salt.
